# Crossed Cerebellar Diaschisis Secondary to Focal Status Epilepticus

**DOI:** 10.1177/19418744241246304

**Published:** 2024-04-06

**Authors:** Jorge Patino, Amy Durand, Grant Meeks, Sri Raghav Sista

**Affiliations:** 1 Department of Neurology, 12339 UTHealth Houston McGovern Medical School, Houston, TX, USA

**Keywords:** seizures, epilepsy, stroke, cerebrovascular disorders, imaging, techniques, cerebellar diseases

**Figure 1.**
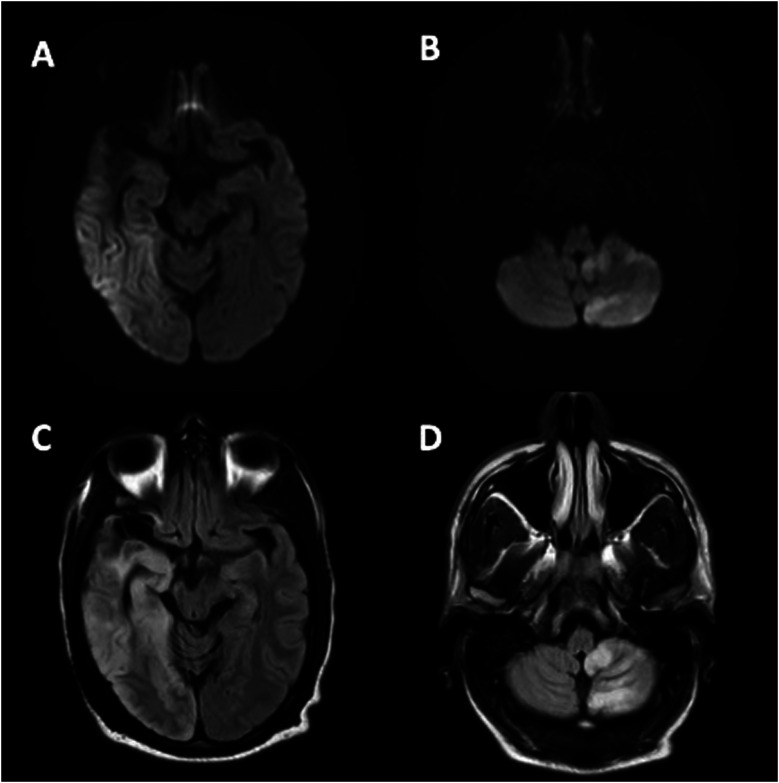
*Brain MRI at presentation*: Restricted diffusion (A), (B) with T2 FLAIR hyperintensities (C), (D) in the right temporoparietal cortex (A), (C) and left cerebellar hemisphere (B), (D).

**Figure 2.**
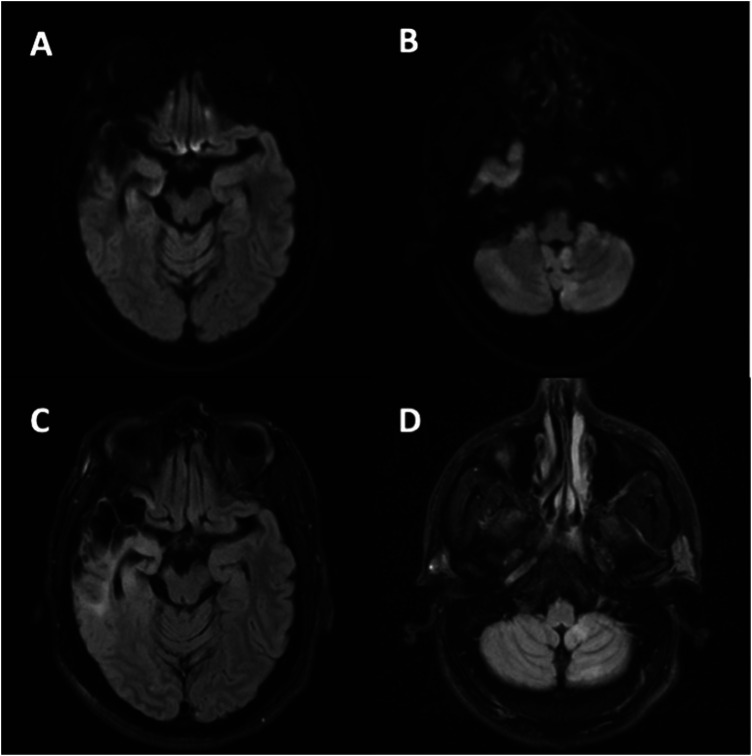
*Repeat Brain MRI 3 days after seizure cessation*: Resolution of previously noted restricted diffusion (A), (B) and T2 FLAIR hyperintensity (C), (D) in the right temporoparietal cortex (A), (C) and left cerebellar hemisphere (B), (D).

A 47-year-old man with chronic ischemic stroke of the right middle cerebral artery (MCA) presented with intractable headache, nausea, and vomiting. Examination revealed fixed left-gaze deviation with retained awareness. Brain MRI displayed T2 FLAIR hyperintensity with restricted diffusion in the right temporoparietal and left cerebellar hemispheres (Figure 1). The electroencephalogram demonstrated right parietal focal status epilepticus. Following the initiation of multiple anti-seizure medications, a subsequent MRI (Figure 2) showed improvement in the findings, suggesting seizures as the reversible cause of cerebellar diaschisis. Crossed cerebellar diaschisis stems from disruption along the corticopontocerebellar tract, inducing contralateral cerebellar hypometabolism after a cortical lesion and causing MRI changes due to possible cytotoxic edema or deafferentiation of second- and third-order neurons.^1^ Frequently observed in MCA stroke, other causative etiologies encompass status epilepticus, Rasmussen’s encephalitis, tumors, etc.^2^ While early intervention, as in our case, can reverse this condition, outcomes are contingent upon the underlying pathology.^3^ When the syndrome is secondary to a chronic lesion such as a stroke, it can cause permanent changes such as hemicerebellar atrophy;^2^ therefore, understanding the imaging might help with an appropriate diagnostic differential.
